# Retraction: Pyridoxal 5′ phosphate protects islets against streptozotocin-induced beta-cell dysfunction – *in vitro* and *in vivo*

**DOI:** 10.3389/ebm.2024.10441

**Published:** 2025-01-09

**Authors:** EBM Editorial Office

Following publication, concerns were raised on the PubPeer platform regarding the reuse of certain images. Particularly, in the β-Actin bands in Figure 6A, two sets of PCR bands appear to be duplicated if reversed horizontally. Further, in the Glut2/Reg-1 bands in the same Figure 6A, two sets of bands appear to be duplicated as well. The authors failed to provide a satisfactory explanation during the investigation, which was conducted in accordance with Experimental Biology and Medicine’s policies. Therefore, the article has been retracted.

**FIGURE 6:**
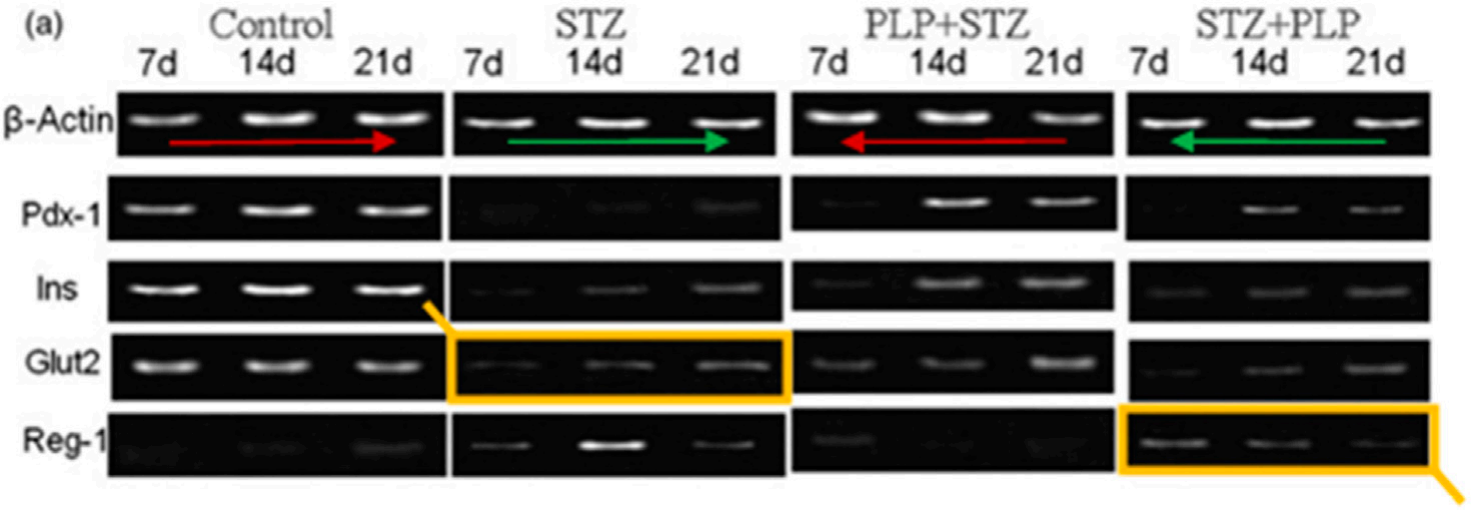
Reverse transcriptase-polymerase chain reaction analysis. **(A)** Expression of Pdx1, Insulin and Glut2 was eventually the same throughout the experimental period in the controls. With STZ treatment the expression of Insulin, Pdx1 and Glut2 was reduced marginally with Reg1 being upregulated also seen in STZ þ PLP group. There was a comparison in the expression of these genes between PLP þ STZ and controls by 21 days.

This retraction was approved by the Editor-in-Chief of Experimental Biology and Medicine. The authors received communication regarding the retraction.

EBM would like to thank the users on PubPeer for bringing the published article to our attention.

