# Erratum: Retraction: Pyridoxal 5′ phosphate protects islets against streptozotocin-induced beta-cell dysfunction – *in vitro* and *in vivo*

**DOI:** 10.3389/ebm.2025.10614

**Published:** 2025-04-24

**Authors:** EBM Production Office

**Affiliations:** Frontiers Media SA, Lausanne, Switzerland

In the published article, there was an error in the terminology used.

A correction has been made to the second and third sentences, which previously stated the following:

“Particularly, in the β-Actin band in **Figure 6A**, two sets of western blots appear to be duplicated if reversed horizontally. Further, in the Glut2 band in the same **Figure 6A**, two sets of western blots appear to be duplicated as well.”

The corrected sentence appears below:

“Particularly, in the β-Actin bands in **Figure 6A**, two sets of PCR bands appear to be duplicated if reversed horizontally. Further, in the Glut2/Reg-1 bands in the same **Figure 6A**, two sets of bands appear to be duplicated as well.”

The publisher apologizes for this error. The original version of this article has been updated.

